# Increases in Health-Related Workplace Absenteeism Among Workers in Essential Critical Infrastructure Occupations During the COVID-19 Pandemic — United States, March–April 2020

**DOI:** 10.15585/mmwr.mm6927a1

**Published:** 2020-07-10

**Authors:** Matthew R. Groenewold, Sherry L. Burrer, Faruque Ahmed, Amra Uzicanin, Hannah Free, Sara E. Luckhaupt

**Affiliations:** ^1^Health Systems and Worker Safety Task Force, CDC COVID-19 Response Team; ^2^Division of Global Migration and Quarantine, National Center for Emerging and Zoonotic Infectious Diseases, CDC; ^3^Division of Field Studies and Engineering, National Institute for Occupational Safety and Health, CDC.

During a pandemic, syndromic methods for monitoring illness outside of health care settings, such as tracking absenteeism trends in schools and workplaces, can be useful adjuncts to conventional disease reporting (*1*,*2*). Each month, CDC’s National Institute for Occupational Safety and Health (NIOSH) monitors the prevalence of health-related workplace absenteeism among currently employed full-time workers in the United States, overall and by demographic and occupational subgroups, using data from the Current Population Survey (CPS).* This report describes trends in absenteeism during October 2019–April 2020, including March and April 2020, the period of rapidly accelerating transmission of SARS-CoV-2, the virus that causes coronavirus disease 2019 (COVID-19). Overall, the prevalence of health-related workplace absenteeism in March and April 2020 were similar to their 5-year baselines. However, compared with occupation-specific baselines, absenteeism among workers in several occupational groups that define or contain essential critical infrastructure workforce^†^ categories was significantly higher than expected in April. Significant increases in absenteeism were observed in personal care and service^§^ (includes child care workers and personal care aides); healthcare support^¶^; and production** (includes meat, poultry, and fish processing workers). Although health-related workplace absenteeism remained relatively unchanged or decreased in other groups, the increase in absenteeism among workers in occupational groups less able to avoid exposure to SARS-CoV-2 (*3*) highlights the potential impact of COVID-19 on the essential critical infrastructure workforce because of the risks and concerns of occupational transmission of SARS-CoV-2. More widespread and complete collection of occupational data in COVID-19 surveillance is required to fully understand workers’ occupational risks and inform intervention strategies. Employers should follow available recommendations to protect workers’ health.

CPS is a monthly national survey of approximately 54,000 households conducted by the U.S. Census Bureau for the Bureau of Labor Statistics. The survey, the nation’s primary source of labor force statistics, collects information on employment, demographic, and other characteristics of the civilian, noninstitutionalized population aged ≥16 years. Data on all sample household members are collected from a single respondent by trained interviewers through in-person or telephone interviews using a standardized questionnaire.^††^

Monthly point estimates and 95% confidence intervals (CIs) of the prevalence of health-related workplace absenteeism among all full-time workers during October 2019 to April 2020 were calculated and compared with an epidemic threshold defined as the upper 95% confidence limit of a historical baseline that represents the expected value and was established using data from the previous 5 years, aggregated by month.^§§^ Estimates with lower 95% confidence limits that exceeded the epidemic threshold were considered significantly higher than expected; this conservative method helps account for multiple comparisons. Comparisons for which the point estimate, but not the lower 95% confidence limit, exceeds the epidemic threshold indicate possible increases and warrant further scrutiny. For such occurrences, the Z-test for independent proportions was used to further test the significance of differences in observed versus expected absenteeism. Results of these post hoc tests with a significance level of p<0.05 were considered equivocal evidence of increased absenteeism. Estimates were also calculated for 22 civilian occupational subgroups^¶¶^ and compared with their occupation-specific epidemic thresholds.

A full-time worker was defined as an employed person aged ≥16 years who reported usually working at least 35 hours per week for all jobs combined. Health-related workplace absenteeism was defined as working <35 hours during the reference week because of the worker’s own illness, injury, or other medical problem. Based on special guidance provided to CPS interviewers by the Bureau of Labor Statistics in March and April 2020, this categorization also applied to persons who indicated they were under quarantine or self-isolating because of exposure to COVID-19.*** Because the CPS questions refer to 1 week of each month, absenteeism during the other weeks is not measured. These 1-week measures are intended to be representative of all weeks of the month during which they occur.

All analyses were weighted using the CPS composite weight and estimates of all standard errors were adjusted to account for the complex design of the CPS sample. Analyses were performed using SAS statistical software (version 9.4; SAS Institute).

During October 2019–February 2020, point estimates of the prevalence of health-related workplace absenteeism among all full-time workers remained at or below the epidemic threshold. In March and April 2020, these estimates exceeded the epidemic threshold, although not significantly (Figure). The Z-test for independent proportions also did not indicate a statistically significant increase in absenteeism in March (p = 0.18) or April (p = 0.06).

**FIGURE:**
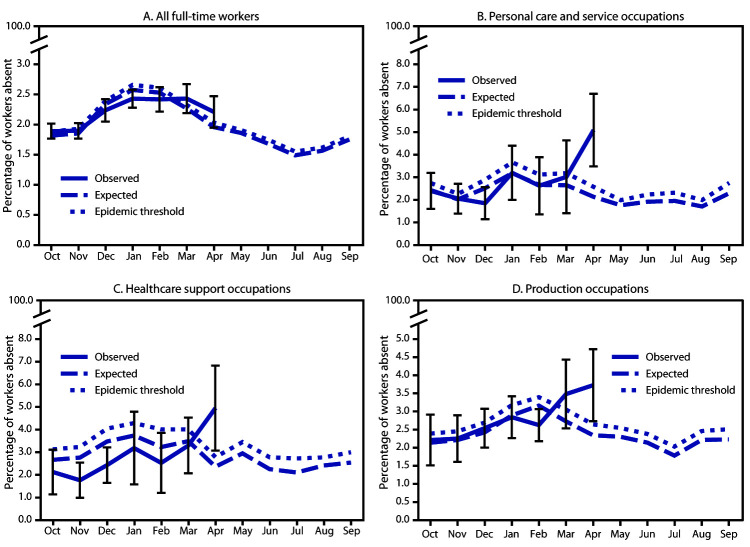
Prevalence* of health-related workplace absenteeism**^†^** reported by full-time workers**^§^** relative to an epidemic threshold,**^¶^** overall (A)** and by occupational subgroup (B, C, D)**^††^****^,^****^§§^****^,^****^¶¶^** — Current Population Survey, United States, October 2019–April 2020 * Error bars represent 95% confidence intervals for point estimates. ^†^ Defined as working <35 hours during the reference week because of illness, injury, or other medical issue. ^§^ Employed persons who usually work ≥35 hours per week at all jobs combined. ^¶^ Epidemic threshold is the upper 95% confidence limit for expected values; expected values are based on monthly averages for the previous 5 years. The expected baseline and epidemic threshold are shown for the entire October–September surveillance period to illustrate expected seasonality. ** All occupations combined. ^††^ Personal care and service occupations include 2010 Census occupation codes 4300–4650. ^§§^ Healthcare support occupations include 2010 Census occupation codes 3600–3655. ^¶¶^ Production occupations include 2010 Census occupation codes 7700–8750.

In April, absenteeism among the following occupational subgroups significantly exceeded their occupation-specific epidemic thresholds based on the nonoverlapping CI criterion: personal care and service, including childcare workers and personal care aides (5.1% [95% CI = 3.5–6.7] observed, versus 2.1% [95% CI = 1.7–2.6] expected); healthcare support (5.0% [95% CI = 3.1–6.8] versus 2.4% [95% CI = 1.9–2.8]; and production, including meat, poultry, and fish processing workers (3.7% [95% CI = 2.7–4.7] versus 2.3% [95% CI = 2.0–2.6]) (Figure) (Table). Based on the Z-test for independent proportions, prevalence in April might also have been higher among transportation and material moving occupations,^†††^ which include bus drivers and subway and streetcar workers (3.6% [95% CI = 2.6–4.6] versus 2.5% [95% CI = 2.2–2.9], p = 0.040), and healthcare practitioner and technical occupations^§§§^ (2.8% [95% CI = 2.0–3.6] versus 1.9% [95% CI = 1.6–2.1], p = 0.017). Absenteeism prevalence either declined or remained flat for all other occupational groups. Absenteeism was not significantly higher than expected for any other group in any month during October 2019–February 2020.

**TABLE:** Monthly prevalence of health-related workplace absenteeism* among full-time workers,^†^ by occupational group — Current Population Survey, United States, October 2019–April 2020

Occupational group	Weighted % (95% CI)						
	Oct–Dec 2019			Jan–Apr 2020			
	Oct	Nov	Dec	Jan	Feb	Mar	Apr
Total	1.9 (1.8–2.0)^§^	1.9 (1.8–2.0)	2.2 (2.0–2.4)	2.4 (2.3–2.6)	2.4 (2.2–2.6)	2.4 (2.2–2.7)^§^	2.2 (1.9–2.5)^§^
Personal care and service	2.4 (1.6–3.2)	2.1 (1.4–2.7)	1.9 (1.1–2.6)	3.2 (2.0–4.4)	2.6 (1.4–3.9)	3.0 (1.4–4.6)	5.1 (3.5–6.7)^¶^
Healthcare support	2.1 (1.1–3.1)	1.8 (1.0–2.5)	2.4 (1.6–3.2)	3.2 (1.6–4.8)	2.5 (1.2–3.9)	3.3 (2.1–4.5)	5.0 (3.1–6.8)^¶^
Production	2.2 (1.5–2.9)	2.2 (1.6–2.9)	2.5 (2.0–3.1)	2.8 (2.3–3.4)	2.6 (2.2–3.1)	3.5 (2.5–4.4)^§^	3.7 (2.7–4.7)^¶^
Transportation and material moving	2.9 (2.1–3.6)^§^	2.2 (1.4–3.0)	2.9 (2.4–3.5)	2.8 (1.8–3.8)	3.1 (2.4–3.8)	3.1 (2.3–3.9)	3.6 (2.6–4.6)**
Building and grounds cleaning and maintenance	1.9 (1.0–2.8)	1.9 (0.9–2.9)	2.9 (2.1–3.8)	2.9 (1.7–4.2)	3.4 (2.4–4.4)	3.2 (1.9–4.5)	3.3 (2.1–4.5)
Food preparation and serving related	2.1 (1.3–2.9)	2.2 (1.3–3.1)	2.7 (1.7–3.6)	2.7 (1.5–3.9)	3.0 (1.9–4.0)	2.8 (1.7–3.8)	3.1 (1.1–5.1)
Construction and extraction	1.4 (0.9–2.0)	1.6 (1.0–2.2)	2.2 (1.7–2.7)	3.1 (2.0–4.1)^§^	2.5 (1.7–3.2)	2.3 (1.4–3.1)	2.9 (1.8–4.1)^§^
Healthcare practitioner and technical	2.3 (1.8–2.8)	2.0 (1.5–2.5)	2.3 (1.7–2.9)	2.4 (1.6–3.2)	2.5 (1.9–3.0)	2.1 (1.5–2.7)	2.8 (2.0–3.6)**
Farming, fishing, and forestry	1.1 (0.0–2.4)	1.4 (0.0–3.5)	1.6 (0.1–3.2)	4.2 (2.1–6.2)^§^	3.7 (0.9–6.5)	2.6 (0.0–5.4)^§^	2.6 (0.0–6.5)
Office and administrative support	2.6 (2.1–3.1)^§^	2.4 (2.1–2.7)	2.7 (2.3–3.1)	3.0 (2.2–3.7)	2.5 (2.1–2.9)	3.0 (2.5–3.5)	2.5 (1.8–3.1)
Legal occupations	2.0 (0.7–3.3)	1.0 (0.1–1.9)	1.5 (0.6–2.5)	2.9 (1.5–4.3)^§^	2.7 (1.0–4.3)	0.9 (0.1–1.8)	2.3 (0.7–3.8)
Sales and related	1.7 (1.3–2.1)^§^	2.1 (1.6–2.7)**	2.0 (1.5–2.6)	2.0 (1.6–2.5)	2.3 (1.5–3.1)^§^	2.1 (1.7–2.6)	2.1 (1.6–2.6)
Protective service	2.7 (1.4–3.9)^§^	2.4 (1.3–3.5)^§^	2.9 (1.6–4.1)	3.3 (2.2–4.3)^§^	2.6 (1.8–3.3)^§^	2.3 (1.6–3.1)	2.1 (1.3–3.0)
Installation, maintenance and repair	2.4 (1.6–3.1)	2.4 (1.6–3.2)	1.9 (1.2–2.6)	1.8 (1.0–2.7)	2.8 (2.1–3.5)	3.5 (2.3–4.7)^§^	2.0 (1.2–2.9)
Education, training, and library	1.5 (1.1–2.0)	2.3 (1.7–2.8)**	2.7 (1.9–3.4)^§^	2.7 (2.1–3.2)^§^	2.5 (1.9–3.0)	2.2 (1.5–2.9)	1.5 (0.8–2.3)
Architecture and engineering	0.8 (0.0–1.7)	1.3 (0.4–2.2)	1.4 (0.6–2.2)	2.5 (1.3–3.6)	1.5 (0.7–2.4)	2.4 (1.3–3.4)^§^	1.4 (0.6–2.1)
Arts, design, entertainment, sports, and media	2.1 (0.7–3.5)	2.1 (0.9–3.3)	2.3 (0.7–3.9)	2.0 (0.7–3.3)	1.6 (0.9–2.4)	2.5 (0.6–4.4)	1.4 (0.3–2.5)
Business and financial operations	1.5 (1.1–2.0)	1.3 (0.7–1.9)	2.1 (1.5–2.6)	2.5 (1.8–3.1)	2.4 (1.9–2.8)^§^	1.6 (0.9–2.2)	1.2 (0.7–1.8)
Computer and mathematical science	1.4 (0.8–2.0)	0.8 (0.3–1.2)	1.6 (0.9–2.2)	1.6 (1.0–2.3)	2.2 (1.3–3.1)	2.0 (1.2–2.8)^§^	1.1 (0.5–1.8)
Community and social service	1.9 (0.7–3.1)	2.5 (1.4–3.6)	1.8 (1.0–2.5)	1.6 (0.8–2.4)	2.3 (1.1–3.4)	3.1 (1.9–4.2)	1.0 (0.0–2.2)
Management	1.1 (0.8–1.4)	1.3 (0.9–1.6)	1.7 (1.4–1.9)	1.3 (1.0–1.6)	1.6 (1.3–1.9)	1.6 (1.3–2.0)	0.9 (0.6–1.2)
Life, physical, and social science	1.9 (0.5–3.4)	2.8 (1.0–4.5)	2.4 (0.8–4.0)	2.9 (1.4–4.4)	2.5 (1.0–3.9)	1.2 (0.3–2.1)	0.5 (0.0–1.2)

**Abbreviation:** CI = confidence interval.

* Defined as working <35 hours during the reference week because of illness, injury or other medical issue.

^†^ Defined as employed persons who usually work ≥35 hours per week at all jobs combined.

^§^ Point estimate, but not its lower 95% confidence limit, exceeded an epidemic threshold defined as the upper 95% confidence limit of the expected value, based on monthly average for the previous 5 years, and p-value for post hoc observed versus expected comparison using Z-test for independent proportion ≥0.05.

^¶^ Significantly exceeded the epidemic threshold (i.e., lower 95% confidence limit of the point estimate exceeded the epidemic threshold).

** Point estimate, but not its lower 95% confidence limit, exceeded the epidemic threshold and p-value for post hoc observed versus expected comparison using Z-test for independent proportion <0.05.

## Discussion

These findings indicate that although the overall impact of the COVID-19 pandemic on health-related workplace absenteeism among full-time workers in March and April 2020 was minor, during April 2020, absenteeism was significantly higher than expected among several occupational groups that either define or contain infrastructure workforce categories deemed essential and critical (health care support occupations, personal care and service occupations, and production occupations) based on their 5-year historical baselines. Many essential critical infrastructure jobs inherently involve prolonged close contact with patients, the general public, or coworkers (*3*). The workers in these occupational groups are also likely to have had to continue to be physically present in their workplaces during March and April and could not avoid exposure by, for example, working from home. For both reasons, workers in these essential critical infrastructure occupations are likely to be at increased risk for occupational exposure to SARS-CoV-2. Equivocal evidence of increased absenteeism in April was found for workers in the transportation and material moving and healthcare practitioner and technical occupations; these occupations are also part of the essential critical infrastructure workforce, and therefore are also likely to be at increased risk for occupational exposure to SARS-CoV-2 for the same reasons.

Health-related workplace absenteeism correlates well with the prevalence of influenza-like illness^¶¶¶^ (*4*), making it a useful measure of the impact of influenza pandemics or seasonal influenza epidemics on the working population (*1*,*2*). Whether this is true of COVID-19 is not yet known. Overall, absenteeism among the employed full-time workforce did not increase in conjunction with the incidence of COVID-19 in March and April; estimates for those months were similar to the 5-year baseline. This finding might be because of increased remote work or telework during these 2 months by those who could do so after implementation of the stay-at-home or shelter-in-place of residence recommendations (*5*), because of workplace control measures implemented to reduce exposures, or because the population most likely to experience symptomatic illness with COVID-19, persons aged >70 years (*6*), did not overlap substantially with the working population. However, the increase in health-related workplace absenteeism specifically among workers in certain occupational groups less able to avoid exposure to SARS-CoV-2 while such absenteeism remained relatively flat or decreased in other occupational groups highlights the potential impact of COVID-19 on the essential critical infrastructure workforce caused by the risks and concerns of occupational transmission of SARS-CoV-2.

The findings in this report are subject to at least seven limitations. First, operationalized, health-related workplace absenteeism includes absences caused by injuries, preventive care, and illnesses unrelated to COVID-19, as well as quarantine-associated absences, which could attenuate or confound absenteeism’s putative relation to COVID-19 incidence. Second, data from the March and April surveys were adversely affected by the pandemic’s impact on the U.S. Census Bureau’s survey operations, resulting in substantial and nonrandom reductions in response rates across respondent groups. However, the Bureau of Labor Statistics was able to obtain estimates that met standards for accuracy and reliability. Third, monthly absenteeism estimates are based on 1-week measures and could have underestimated or overestimated the actual prevalence for any given month in a way that is not reflected in the 95% CIs. Fourth, the nature of the CPS data only allows for calculation of health-related absenteeism among full-time workers; patterns of absenteeism might be different among part-time workers. Fifth, the occupational subgroups analyzed include multiple occupations with heterogeneous levels of exposure to patients, clients, or members of the public with COVID-19. Sixth, prevalences of absenteeism in this report are not adjusted to control for the effect of potential sociodemographic confounders such as age, sex, race, or ethnicity. Finally, these national analyses might have failed to detect localized increases in absenteeism in specific geographic regions.

These findings are consistent with those from public health surveillance and field investigations suggesting that certain groups of workers might be at increased risk for SARS-CoV-2 infection because of their work during the pandemic, including health care personnel (*7*,*8*) and food production workers (*9*), among others (*10*). CDC and Occupational Safety and Health Administration guidance for protecting essential critical infrastructure workers is available and should be followed by their employers.**** In addition, improved surveillance is needed to monitor industry-specific and occupation-specific morbidity and mortality in this and future pandemics. In May 2020, CDC revised its COVID-19 Case Report Form to record certain health care–specific occupations, as well as limited information on suspected workplace exposures and settings for essential critical infrastructure workers.^††††^ Collection of additional information on work characteristics^§§§§^ might help better describe the occupational risk and impact of COVID-19 and inform intervention strategies.

**Summary**

**What is already known about this topic?**

Syndromic methods for monitoring illness outside health care settings, such as tracking absenteeism trends in schools and workplaces, can be useful adjuncts to conventional disease reporting in the pandemic setting.

**What is added by this report?**

Whereas the overall impact of COVID-19 on health-related workplace absenteeism in March and April was minor, increases in absenteeism in personal care and service, healthcare support, and production occupations, groups that contain or define essential critical infrastructure workforce categories, highlight the risks and concerns surrounding occupational transmission of SARS-CoV-2.

**What are the implications for public health practice?**

Collection of additional occupational data in COVID-19 surveillance might help better understanding of the occupational risk and impact of COVID-19 and identify intervention opportunities.

## Footnotes

* https://www.cdc.gov/niosh/topics/absences/default.html.

† https://www.cisa.gov/sites/default/files/publications/Version_3.0_CISA_Guidance_on_Essential_Critical_Infrastructure_Workers_1.pdf.

§ Includes 2010 Census occupation codes 4300–4650. Examples of personal care and service occupations include childcare workers; personal and home care aides; barbers; hairstylists and cosmetologists; recreation and fitness workers; morticians; embalmers; and porters and bellhops.

¶ Includes 2010 Census occupation codes 3600–3655. Examples of healthcare support occupations include nursing, psychiatric, and home health aides; various therapy aides and assistants; medical and dental assistants; and phlebotomists.

** Includes 2010 Census occupation codes 7700–8750. Examples of production occupations include assemblers and fabricators; food processing workers; metal and plastic workers; printing workers; textile, apparel, and furnishing workers; woodworkers; and plant and system operators.

†† https://www.census.gov/programs-surveys/cps.html.

§§ https://www.cdc.gov/flu/weekly/overview.htm.

¶¶ Occupational subgroups correspond to the CPS Detailed Occupational Group recodes, which are groupings of Census occupation codes (https://www2.census.gov/programs-surveys/cps/methodology/Occupation%20Codes.pdf). The Census occupation codes are, in turn, based on the Bureau of Labor Statistics 2010 Standard Occupational Classification codes (https://www.bls.gov/soc/2010/home.htm).

*** https://www.bls.gov/cps/employment-situation-covid19-faq-march-2020.pdf; https://www.bls.gov/cps/employment-situation-covid19-faq-april-2020.pdf.

††† Includes 2010 Census occupation codes 9000–9750. Examples of transportations and material moving occupations include transportation workers, such as bus and taxi drivers, and material moving workers, such as crane operators and hand packers and packagers.

§§§ Includes 2010 Census occupation codes 3000–3540. Examples of healthcare practitioners and technical occupations include health diagnosing and treating practitioners, such as physicians, dentists, pharmacists, and nurses, and health technologists and technicians, such as dental hygienists, radiologic technicians, and paramedics.

¶¶¶ Fever (temperature of ≥100°F [37.8°C) and a cough and/or sore throat without a known cause other than influenza.

**** https://www.cdc.gov/coronavirus/2019-ncov/community/worker-safety-support/index.html.

††††
https://www.cdc.gov/coronavirus/2019-ncov/php/reporting-pui.html .

§§§§
https://www.cdc.gov/niosh/topics/coding/ .
